# Problematic Smartphone Use and Problematic Social Media Use: The Predictive Role of Self-Construal and the Mediating Effect of Fear Missing Out

**DOI:** 10.3389/fpubh.2022.814468

**Published:** 2022-02-23

**Authors:** Rocco Servidio, Beatrix Koronczai, Mark D. Griffiths, Zsolt Demetrovics

**Affiliations:** ^1^Department of Cultures, Education and Society, University of Calabria Arcavacata di Rende, Cosenza, Italy; ^2^Department of Developmental and Clinical Child Psychology, Institute of Psychology, ELTE Eötvös Loránd University, Budapest, Hungary; ^3^International Gaming Research Unit, Psychology Department, Nottingham Trent University, Nottingham, United Kingdom; ^4^Centre of Excellence in Responsible Gaming, University of Gibraltar, Gibraltar, Gibraltar; ^5^Department of Clinical Psychology and Addiction, Institute of Psychology, ELTE Eötvös Loránd University, Budapest, Hungary

**Keywords:** self-construal, fear of missing out (FoMO), problematic smartphone use (PSU), problematic social media use (PSMU), interdependent self-construal, independent self-construal

## Abstract

Problematic smartphone use (PSU) and problematic social media use (PSMU) are two interrelated constructs which have received significant research attention over the past decade. The present study investigated the relationship between self-construal (distinguished as independent and interdependent), PSU and PSMU with Fear of Missing Out (FoMO) as a mediating variable. The sample comprised 405 Italian students who completed standardized psychometric scales assessing the variables of the study. Bivariate correlations analysis showed that FoMO and independent self-construal was significantly and negatively associated. On the contrary, interdependent self-construal was significantly and positively associated with FoMO, PSU, and PSMU. Mediation analysis showed that FoMO mediated the relationship between self-construal and both PSMU and PSU, but at different levels. The results demonstrated that FoMO full mediated the relationships between interdependent self and PSU, whereas only partial mediation was found between interdependent self and PSMU. Therefore, taking these personality characteristics into account may help reduce dysfunctional behaviour associated with problematic technology use and promote psychological well-being among students. However, it is recommended that further studies replicate the proposed model by including other psychological constructs.

## Introduction

The powerful combination of internet and mobile technologies has increased the risk of new behavioral addictions. One of these potential behavioral addictions, namely problematic smartphone use (PSU), has emerged as a phenomenon of increased academic concern. PSU refers to excessive use of a smartphone with accompanying functional impairments in daily living, and symptoms resembling those found in more traditional psychoactive substance use disorders (1). Additionally, it is important to investigate not only the consequences of excessive use but the predisposing factors and specific use motives, as well as cognitive and affective variables (e.g., expectancies, experienced gratification), leading to the problematic overuse of specific mobile applications (2).

Cross-sectional analysis has shown that PSU behavior has been associated with different negative consequences, such as depression (3), poor sleep quality [(4), for a review], poor academic achievement (5), and loss of control (6, 7). According to an international report, 97% of Italian individuals used their smartphone to access the internet and about 85.2% has been actively engaged with or contributed to social media in terms of communication among social groups (8). A recent meta-analysis identified specific predictive factors for PSU [(9), for a review]. Among these risk factors, it was found that individuals with a large social network may spend more time on smartphones than others to maintain relationships, increasing the risk of PSU over time.

A recent study conducted by Canale et al. (10), which used a Bayesian analyses approach, provided the first comprehensive test of the pathway model of PSU as posited by Billieux et al. (1). The results of the study indicated that each path (i.e., excessive reassurance, impulsive, and extraversion), was associated with distinct psychosocial and psychopathological variables. Therefore, several factors can influence the development of PSU. Among these factors, personality traits such as low self-control (7), Dark Triad traits (11, 12), and self-esteem (13, 14), as well as the contribution of metacognitions and smartphone use expectancies (15) have been identified as significant predictors of PSU. Moreover, personality is one of the most investigated variables since individual differences can shed light if specific personality traits predispose technology users to develop addictive behavior. The results of a recent meta-analysis indicated the presence of robust associations between PSU and high levels of neuroticism and lower levels of conscientiousness [(16), for a review].

Although PSU and problematic social media use (PSMU) are not included in the latest fifth edition of the *Diagnostic and Statistical Manual of Mental Disorders* (DSM-5), research concerning these new behavioral addictions should be addressed to understand the motivational factors and the clinical features, as well as the co-occurrence with other disorders rather than overemphasize specific technological aspects (i.e., stressing a dichotomy between predominantly mobile and predominantly non-mobile internet-use disorder) (17). PSMU can be described as an unhealthy excessive form of social media use, which is characterized by a lack of control over the behavior and continued behavior despite clinically impairing life consequences [see (18, 19), for a review]. Social media platforms (i.e., *Facebook, Instagram, Twitter*, etc.) are typically available over mobile devices with an internet connection. However, for a minority of individuals, the use of social media applications can become problematic or pathological, interfering with daily functioning, social relationships, and academic or work performance (20). Additionally, social media use is one of the most predominant activities engaged in on smartphones (21, 22), and use of social media is considered a vulnerability factor in developing PSU (23, 24). Smartphones with internet access provide constant availability to social media applications and provides immediate access for individuals to check their notifications, especially among those with lower self-control, which may result in PSU and PSMU [see (7, 24), for a review]. Consequently, it is important to explore possible psychological factors that may increase the risk of PSU and PSMU.

Markus and Kitayama (25) developed self-construal theory, which can be described as the “belief about the relationship between the self and others and, especially, the degree to which they see themselves as separate from others or as connected with others” (p. 226). Subsequently, Singelis (26) conceptualized self-construal “as a constellation of thoughts, feelings, and actions concerning one's relationship to others, and the self as distinct to others” (p. 581). More specifically, self-construal theory distinguishes between independent self and interdependent self. Individuals with independent self-construal evaluate themselves not in the context of others, such as family members, co-workers or colleagues, but are more self-contained and autonomous (27). In this view, the self is separate from the social context. More specifically, when individuals think about themselves, those with highly independent self-construal will have their abilities and attributes as a referent rather than referring to the thoughts or actions of others (28). This suggests they have a high level of self-esteem which allows them to validate their internal attributes. Conversely, individuals with interdependent self-construal feel more connected with others. Therefore, individuals are not separated from the social context and are more closed and less differentiated from others.

Individuals can develop both independent self and interdependent self simultaneously. Therefore, in everyone, these two aspects of self can coexist. For this reason, it is not correct to consider independent and interdependent self-construal as two extremes of the same dimension. They should be conceptualized as different aspects of the self-concept. Indeed, some individuals might have an independent self or an interdependent self that is more salient (25). This view avoids categorizing individuals as having either an independent self or interdependent self, rather individuals should be described as having a salient interdependent or independent self-construal (27, 29).

Previous findings on self-construal have demonstrated that it is an influential psychological variable that affects human technologies interaction as well as social media use (30). For example, a review of the literature suggested that individuals with an interdependent self are more prone to use social media (31). The results of another study showed that individuals with interdependent self-construal were more disposed to share personal information on online social media (32). Recently, the results of another study reported that females, compared to males, exhibited stronger prosocial motives for using SNSs, which was explained by their interdependent self-construal (33). Moreover, in their research on private university students in Lebanon, Hawi and Samaha (34) found that only independent self-construal was a significant and negative predictor of PSMU. However, as a personality trait, self-construal has been explored only with PSMU, but has not been widely considered in relation to its association with PSU (35). Therefore, investigating whether or not self-construal affects PSU is one of the objectives of the present paper.

Additionally, the present study investigated whether fear of missing out (FoMO) would significantly mediate the associations between self-construal, PSMU, and PSU. The concept of FoMO describes the individual's desire to keep up with what other individuals are doing online as well as the idea that other individuals experience more interesting events when the user is not online (36). Previous researchers have shown that FoMO can contribute to both PSMU (37) and PSU [see (37), for a review]. These findings are important to understand the association between the variables in the present study. A recent meta-analysis reported a significant positive relationship between FoMO and both social media use and PSMU (38). The apprehension that others might be having gratifying experiences from which an individual is absent seems to be one of the contributory factors in PSMU, driven by the need to get in touch with others, mainly when individuals use social media applications to socially interact. Therefore, FoMO appears to be a strong motivator underlying PSMU. It has been indicated that social media use and FoMO have a reciprocal relationship, and FoMO drives PSMU to fulfill individual psychological needs of belongingness, consequently reinforcing the risk of PSU because social media applications are mainly used by individuals on their smartphones (39, 40).

Moreover, prior studies have explored the mediating role of FoMO, supporting the proposed argument. For example, a recent study demonstrated that FoMO mediated the relationship between maximization (a personality trait that describes individual differences in the general tendency of striving to make the best choice) and PSU (13). Furthermore, the extant literature has demonstrated that FoMO can mediate psychological variables (i.e., depression, envy) and PSU (3, 41). A survey study also found that FoMO and relational (interdependent) self-construal had a moderated mediation effect on the relationship between smartphone addiction and interpersonal sensitivity (i.e., a personality tendency characterized by constantly worrying about negative social evaluation) (35). Although FoMO has been conceptualized in the framework of the self-determination theory (36), the relationship between self-construal and FoMO is still unclear. However, the findings of a recent study provided consistent and convergent findings that FoMO is positively associated with interdependent self-construal (30). Consequently, individuals with a salient interdependent self, compared to those with a salient independent self, may experience higher levels of FoMO. Indeed, individuals with salient interdependent self-construal evaluate other individuals around them as a part of their identities (42) and will be more worried about what other individuals around them are doing, especially when they are included in online social groups. Therefore, if individuals evaluate other people as a part of themselves, the likelihood of experiencing FoMO would be higher, especially for those who do not feel socially close to others in the offline world (43). Moreover, daily frustration to satisfy basic psychological needs may lead to high levels of FoMO, and in turn could motivate individuals to constantly engage with mobile social media applications subsequently increasing the risk of developing PSMU and PSU (44).

Individuals with a salient independent self, usually, feel less connected to others, since they do not expect to receive social approval (i.e., likes and positive remarks from their friends). Conversely, individuals with a salient interdependent self are more likely to fear that they could be missing out on things with their friends on social media platforms when temporarily leaving the social applications, which can lead to higher levels of FoMO. In other words, the independent self should be negatively related to FoMO. On the other hand, the salient interdependent self should be positively associated with FoMO. This view is supported by a previous study, which indicated that FoMO was significantly higher among individuals with low levels of self-esteem (13). However, no previous studies have explored how FoMO works in these relationships. Furthermore, a growing number of studies indicate that FoMO is positively and significantly associated with both PSMU and PSU (45–47). Additionally, a cross-sectional study indicated that FoMO is more important than other variables (e.g., avoidant attachment and anxiety) in predicting PSMU (48). Moreover, when controlling for FoMO, the effect between some personality traits (e.g., extraversion, and neuroticism), and PSMU no longer becomes significant because only FoMO predicts PSMU. Therefore, the associations between self-construal, PSMU, and PSU may be mediated by FoMO.

Relevant to the present study is the Interaction of Person-Affect-Cognition-Execution (I-PACE) model (49), which is a comprehensive model of factors influencing internet use/overuse. Personal factors, for example, include genetic and biological influences, psychopathology, personality, cognitions, and use motives. Responses to such personal factors comprise mechanisms that may be risk or resilience factors for internet use, including cognitive biases, coping style, etc. Such responses may lead to the decision to use a particular type of internet-enabled device (e.g., smartphone) or use a specific social media platform, which may lead to healthy gratification or excessive use (50). Additionally, according to Marengo et al. (16), the I-PACE model provides a theoretical framework to study internet-related disorders since there is an overlap between PSMU and PSU. Therefore, the present study investigated the relationships between self-construal, PSMU, and PSU, with FoMO as a mediating variable. As aforementioned, self-construal theory proposes self-independent and interdependent dimensions. In particular, the interdependent self is based on the individual's connection to others, demonstrating that the ability to fit into social groups represents an important basis of increasing self-esteem. At early stages, focusing on the person component of the I-PACE model (51), it has been found that personality traits, such as low conscientiousness, low self-esteem, and high neuroticism, are factors driving a heightened risk for internet-use disorders. Therefore, it is argued that individuals with interdependent self would be more concerned with what others are doing, which could result in high levels of FoMO. In the I-PACE model, FoMO would be categorized as a response style and involves negative mood in which individuals attempt to regulate by using technological devices such as smartphones or internet applications like social media allowing individuals to escape from daily stressful issues.

## Hypotheses of the Present Study

Given the diffusion of smartphones and the adverse influence on the self, it is important to explore the role of mediating mechanisms which can help in the development of prevention and intervention programs. Therefore, the primary objective of the present study is to examine why some individuals spend more time online on social media and use their smartphones so excessively that it results in symptoms of behavioral addiction (43). To address this research objective, it was hypothesized that individuals who view themselves as socially connected with others, missing any social event or discussion (interdependent self) will generate feelings of FoMO (H1), and in turn increase the risk of technological addiction (PSMU and PSU) (H2). However, if PSU is associated with different factors, such as cognition, personality, existing psychopathology, and motives for internet-related applications and smartphone use, as suggested by I-PACE model, dealing with these factors leads individuals to use social media platforms excessively could help individuals with PSU. Therefore, it was hypothesized that FoMO would mediate the predictive effect of self-construal on PSMU and PSU (H3). Finally, age and gender were entered as covariates of PSMU and PSU, since prior studies have reported younger age and female gender as significant predictors of PSMU (52) and PSU (53).

## Methods

### Participants and Procedure

Participants were recruited on the university campus during regular teaching activities. After obtaining their informed consent, all participants were introduced to the study objectives, and they were invited to complete an anonymous and confidential paper-pen-pencil survey. All participants volunteered for the study and none of them received any kind of remuneration. Moreover, they were also allowed to withdraw their data from the study at any stage. Completing the questionnaire took ~25 min. The language of the questionnaire was Italian. All the research material and procedures were designed according to the guidelines laid out by Ethics in Human Research and the Italian Association of Psychology. Approval for the study was provided by the Ethics Committee of the University of Calabria (Prot. n. 0043310).

The sample comprised 405 Italian students attending various university degrees courses (114 males [28.15%] and 288 females [71.11%]). Three students did not report their gender. The participants' ages ranged from 19 to 43 years (M = 22.11 years, SD = 3.80). Most participants were attending psychological and educational courses (60.48%). The remainder were enrolled on various courses such as economics (10.86%), engineering (5.18%), mathematics (8.39%), and computer science (11.85%). The remaining students (3.21%) did not indicate their degree courses.

### Measures

#### Self-Construal

The 10-item Italian version of the Self-Construal Scale [(54), original version (26)] was used to assess two dimensions of the self: independent self-construal (e.g., “*I do my own thing, regardless of what others think”*), and interdependent self-construal (e.g., “*My happiness depends on the happiness of those around me”*). Participants report their level of agreement with each statement on a seven-point scale ranging from 1 (*strongly disagree*) to 7 (*strongly agree*). Scores range from 10 to 70 with higher total scores indicating higher levels of self-construal. The independent self-construal (α = 0.64) and interdependent self-construal (α = 0.65) subscales in the present study demonstrated lower than desired reliabilities (55).

#### Fear of Missing Out (FoMO)

The 10-item Italian version of the FoMO Scale [(56), original version (57)] was used to assess disposition toward FoMO. The scale items (e.g., “*I fear others have more rewarding experiences than me”*) are rated on a five-point scale from 1 (*not at all true of me*) to 5 (*extremely true of me*). Scores range from 10 to 50 with higher total scores indicating a higher level of FoMO. The reliability of the scale in the present study was good (α = 0.73).

#### Problematic Social Media Use (PSMU)

The six-item Italian version of the Bergen Social Media Addiction Scale (BSMAS) [(58), original version (59)] was used to assess the risk of social media addiction over the past year. The six scale items (e.g., “*How often during the last year have you tried to cut down on the use of social media without success?”*) each reflect core addiction elements [i.e., salience, mood modification, tolerance, withdrawal, conflict, and relapse; (51)] and are rated on a five-point scale from 1 (*very rarely*) to 5 (*very often*). Scores range from 6 to 30 with higher scores indicating a greater risk of problematic social media use. The reliability of the scale in the present study was just below what would be ideally desired (α = 0.69).

#### Problematic Smartphone Use (PSU)

The 10-item Italian short version of PSU Scale [(60), original version (57)] was used to assess problematic smartphone use. The items (e.g., “*I have used my smartphone longer than I had intended”*) are rated on a six-point scale from 1 (*strongly disagree*) to 6 (*strongly agree*). Scores range from 10 to 60 with higher scores indicating higher problematic smartphone use. The scale's reliability in the present study was very good (α = 0.80).

### Statistical Analyses

Preliminary statistical analyses were carried out with IBM SPSS Statistics v. 26 software. Descriptive analyses and bivariate correlations were computed. The mediating model was tested where FoMO was inserted as a mediator in the relationship between self-construal (predictor) and PSMU and PSU (outcomes). The mediational analysis was conducted *via* the use of the bootstrapping method with 95% bias-corrected confidence intervals and 10,000 bootstrap samples. The mediation model was estimated with Mplus 7.04 (61).

## Results

### Preliminary Results

Descriptive statistics and bivariate correlations of the main variables are shown in Table 1. The results indicated that individuals with high interdependent self were more likely to have high levels of PSU. However, no significant association was found between independent self and PSU.

**Table 1:** Descriptive statistics and correlation coefficients between study variables.

	***M***	***SD***	**Skewness**	**Kurtosis**	**1**	**2**	**3**	**4**	**5**	**6**	**7**
Independent self	4.95	1.02	−0.19	−0.41	–						
Interdependent self	3.79	0.99	0.05	−0.17	0.06	–					
FoMO	2.17	0.59	0.61	0.32	−0.16^**^	0.12^*^	–				
PSMU	2.10	0.68	0.57	−0.03	−0.02	0.16^**^	0.35^***^	–			
PSU	2.68	0.85	0.35	−0.20	−0.05	0.14^**^	0.35^***^	0.58^***^	–		
Age	22.11	3.65	0.12	6.17	0.02	−0.02	−0.13^**^	−0.14^**^	−0.02	–	
Gender	–	–	–	–	0.03	−0.02	−0.06	0.14^***^	0.12^*^	−0.6	–

*FoMO, fear of missing out; PSMU, problematic social media use; PSU, problematic smartphone use. Gender (0 = male, 1 = female) is point serial correlation (r_pb_)*.

* *p < 0.05;*

** *p < 0.01;*

*** *p < 0.001*.

The mean of the self-construal scale indicated tendencies toward independent self rather than interdependent self in the present sample. Therefore, all the correlations values between the constructs were significant and satisfied the conditions for performing the mediation analysis.

### Mediation Analysis

**Figure 1:**
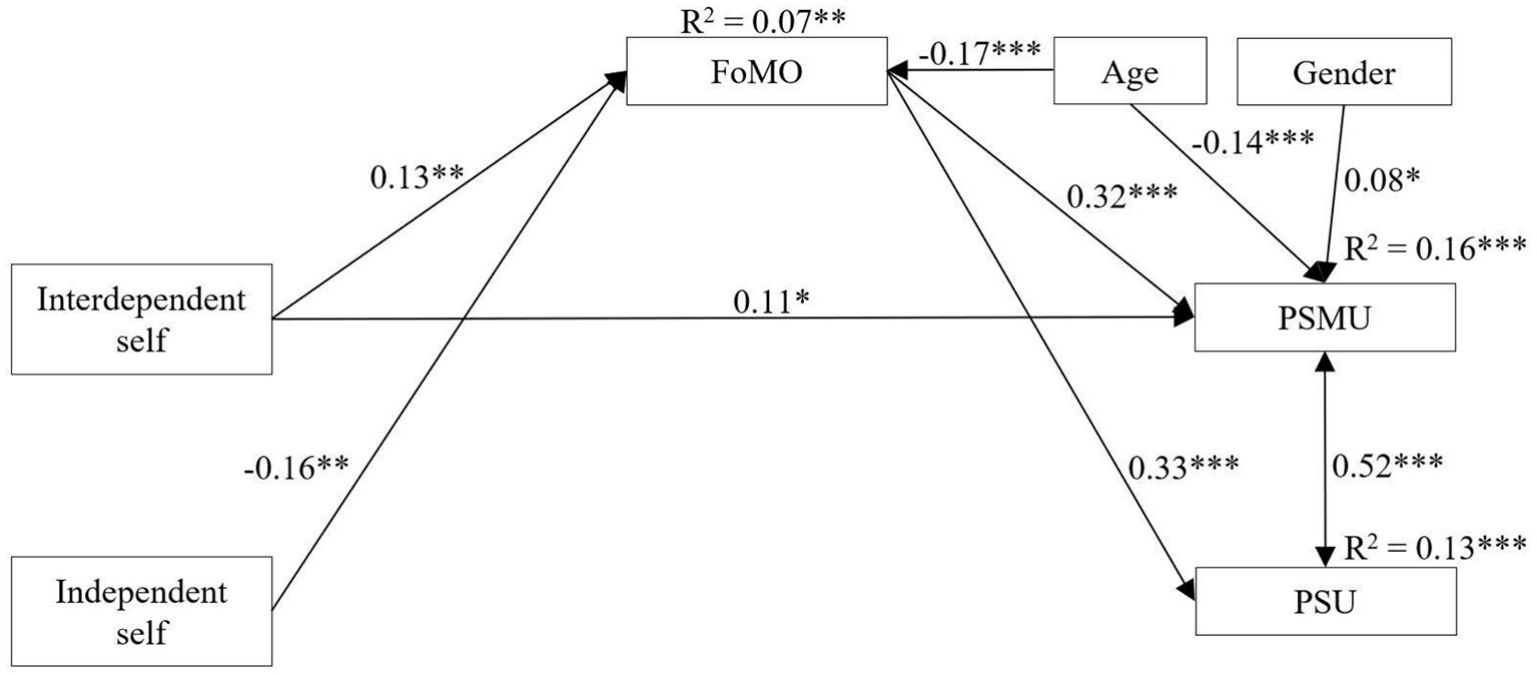
Final estimated path model. For clarity, only the significant relationships are depicted in the figure. FoMO, fear of missing out; PSU, problematic smartphone use; PSMU, problematic social media use. *p < 0.05; **p < 0.01; ***p < 0.001.

The results of the bootstrapping mediational analysis (Figure 1), controlled for gender and age, showed that the independent self was weakly negatively related to FoMO (β = −0.160, *SE* = 0.050, *t* = −3.17, *p* < 0.01, 95% CI [−0.26, −0.06]), while interdependent self was weakly positively related to FoMO (β = 0.129, *SE* = 0.046, *t* = 2.82, *p* < 0.01, 95% CI [0.04, 0.22]), and PSMU (β = 0.110, *SE* = 0.049, *t* = 2.24, *p* < 0.05, 95% CI [0.01, 0.21]). FoMO was positively associated with PSMU (β = 0.322, SE = 0.049, *t* = 6.61, *p* < 0.001, 95% CI [0.22, 0.42]) and PSU (β = 0.335, *SE* = 0.048, *t* = 7.04, *p* < 0.001, 95% CI [0.24, 0.42]), respectively. Additionally, there was no significant association between interdependent self and PSU (β = 0.094, *SE* = 0.049, *t* = 1.91, *p* = 0.056, 95% CI [−0.15, 0.04]). Age was negatively associated with FoMO (β = −0.173, *SE* = 0.048, *t* = −3.60, *p* < 0.001, 95% CI [−0.26, −0.07]) and PSMU (β = −0.138, *SE* = 0.040, *t* = −3.49, *p* < 0.001, 95% CI [-0.22,−0.06]), respectively. On the other hand, gender was only associated with PSMU (β = 0.079, *SE* = 0.036, *t* = 2.15, *p* < 0.05, 95% CI [0.00, 0.15]). Regarding the indirect effects (Table 2), FoMO partially mediated the relationship between interdependent self and PSMU. Finally, a full mediated effect of FoMO was found in the association between interdependent self and PSU.

**Table 2:** Mediation and indirect effects with standardized estimates of FoMO for the relationship among self-construal (independent self and interdependent self), PSMU, and PSU.

**Pathway**	***Estimate***	***SE***	***Z***	***p***	**95% [CI]**
**Independent self** ** → PSMU**					
Total	−0.03	0.05	−0.49	0.621	[−0.13, 0.08]
Indirect	−0.05	0.02	−2.89	0.004	[−0.09, −0.02]
Direct	−0.03	0.05	0.52	0.602	[−0.07, 0.12]
**Independent self** ** → PSU**					
Total	−0.06	0.06	−1.14	0.256	[−0.17, 0.05]
Indirect	−0.05	0.02	−2.90	0.004	[−0.09, −0.02]
Direct	−0.01	0.05	−0.19	0.849	[−0.11, 0.10]
**Interdependent self** ** → PSMU**					
Total	0.15	0.05	3.11	0.002	[0.05, 0.24]
Indirect	0.04	0.02	2.59	0.010	[0.01, 0.08]
Direct	0.11	0.05	2.27	0.025	[0.01, 0.21]
**Interdependent self** ** → PSU**					
Total	0.14	0.05	2.72	0.007	[0.04, 0.23]
Indirect effect	0.04	0.02	2.58	0.010	[0.01, 0.08]
Direct	0.09	0.05	1.91	0.056	[−0.00, 0.18]

*SE, standard error; PSMU, problematic social media use; PSU, problematic smartphone use*.

Table 2 shows the results of the mediation analysis. The only significant effect was the relationship between interdependent self and PSMU, which was partially mediated by FoMO. FoMO fully mediated the relationship between self-construal and PSU. No other mediating effects emerged. Finally, the direct relationships between independent self on both PSMU and PSU were not significant.

## Discussion

The present study examined the mediating role of FoMO in understanding the relationship between self-construal (interdependent self and independent self), PSMU, and PSU both as to outcome variables. It was hypothesized that individuals with higher levels of interdependent self-construal would experience feelings of FoMO (H1), and in turn would be more at risk of PSMU and PSU (H2) both directly and indirectly through the mediating role of FoMO (H3). Overall, the present results suggest that interdependent self-construal was positively related to FoMO, which in turn was associated with greater PSMU and PSU (40). Consistent with the assumptions of the I-PACE model (49) FoMO, which can be considered a maladaptive psychological status, led to both PSMU and PSU. The results of the present paper contribute to the literature on FoMO by demonstrating that interdependent self is positively related to FoMO, and that independent self is negatively associated with FoMO, supporting the present authors' assumptions. Therefore, the present study contributes to the literature on behavioral addiction, particularly in relation to the mediating role of FoMO, by proposing a theoretical framework based on the self-construal theory (25). According to Dogan (30) when a person develops a self-concept of which other individuals are a part, a person will be more inclined to wonder about what others are doing. Individuals develop and shape themselves in connection with others, which represent a part of themselves, and then the desire to stay continually connected with what others are doing becomes inevitable (42, 43). Individuals with an interdependent self-construal are more likely to value connectedness (29). Consequently, they might experience FoMO due to the perceived disconnect with other individuals around them.

Moreover, FoMO could be one of the symptoms of individuals with an interdependent self who are struggling with problems of loneliness. These results are consistent with cross-sectional and experimental studies [e.g., (62)]. Compared to individuals with an independent self, those with a higher interdependent self would prefer social media over face-to-face relationships because this allows them to validate their internal attributes, and subsequently might lead to PSMU. Therefore, individuals with higher levels of interdependent self-construal are more worried about being negatively evaluated by others and are more afraid of missing the latest news concerning others, and are consequently more prone to use mobile social media applications excessively (31).

These results could explain why some types of individuals would be less concerned with active social media use, which in turn are more at risk in the use of smartphone applications. Individuals with a salient interdependent self may repeatedly experience FoMO. They may give less importance to their daily experiences, which represent an important opportunity to shape their sense of self, due to the inner desire to track others' everyday experiences using social media platforms. This allows them to regulate their levels of social appraisal and self-promotion with others. FoMO could work as an internal control mechanism for individuals with a salient interdependent self by offering the ability to protect their well-being aiming to seek reassurance and self-worth (30).

Viewed from the perspective of cultural factors, studies have shown that individuals living in a collectivistic culture tend to develop the interdependent self and report lower life satisfaction compared to individuals from individualistic cultures (30, 34). The present study reinforces these previous findings and underlines the importance of considering the role of FoMO in the relationship between the cultural aspects regarding self-construal theory and internet-related disorders in future cross-cultural psychology studies, especially because FoMO becomes a key factor when individuals in construing themselves give more importance to their connection to the community.

Consistent with the study's hypotheses, it was found that FoMO was a mediator in the relationship between self-construal and PSMU and PSU, but at different levels. The study found a fully-mediated relationship between interdependent self and PSU, and a partially-mediated association between interdependent self and PSMU. According to the results, the effects of FoMO leading to PSMU and PSU by considering the levels of self-construal were different. Interdependent self would reinforce FoMO, which in turn facilitates both PSMU and PSU among university students. Therefore, FoMO is not only an outcome that is affected by self-construal but also an internal motivation of PSMU and PSU behavior. These results fit with the I-PACE conceptualization of cognitive bias and coping style because of mechanisms in the relationship between predisposing factors and excessive internet-use disorders and related applications.

On the other hand, the negative association of independent self with FoMO indicates that people who perceive themselves as separate from others would be less concerned with active use of social applications for social purposes, and therefore they have less risk to develop addictive behavior to technologies (34). These differences in FoMO can also have implications in understanding the existence of indirect paths between self-construal on both PSMU and PSU. Additionally, the results of the present study underline that there are differences in the predictive and mediating variables of both PSMU and PSU. Therefore, these findings are important for designing intervention and prevention programs. Given the widespread use of both smartphones and social media applications, future preventive activities could be designed to promote the adoption of adaptive strategies to manage the experience of FoMO and consequently the risk of developing internet-related disorders. Intervention and prevention programs could focus on helping smartphone and social media users engage in more physical activities and develop offline social networks focusing on face-to-face interactions rather than through technology-mediated environments such as social media platforms (9).

Overall, the strategy that individuals adopt to develop their self-concept affects FoMO. When individuals use social media *via* smartphone applications to satisfy their psychological needs, FoMO will be more salient and the risk of developing an addiction to PSMU, and PSU is higher. Therefore, individuals should focus on personal autonomy to refrain from experiencing the FoMO, which should protect them from the negative consequences of the FoMO because it eases the awareness of the experience that the individual is missing out (30, 63). These results provide insight on the underlying mechanisms between self-construal, where the self is one of the core elements of an individual's identity, and internet-related disorders such as PSMU and PSU. Moreover, the results may also support the I-PACE model, which underlines that addictive behaviors (i.e., PSMU, PSU) result from interactions between a person's core characteristics and mediating/moderating variables (49), which may be dynamic and develop over time because of engagement in specific behaviors likely to be experienced as rewarding because it may help relieve stress.

The present study is not without limitations. First, although path analysis was used to test the study's hypotheses, the study was correlational. Therefore, no causal relationships can be inferred. Researchers should perform longitudinal studies to confirm the inferred causal relationships in the present study. Second, the data in the present study were all self-report. Therefore, future research could benefit by collecting data using mixed methods research approaches. Third, the present study only collected data from Italian university students with a bias toward females, therefore, the generalization of the findings is limited. Future research should use more diverse and representative samples to confirm these findings. Finally, the Cronbach alpha reliability values of the subscales in the Self-Construal Scale were below 0.7 which were less than ideal. Future studies should examine the proposed research model by considering the cultural-related and individual differences, for instance, between Asian and Western individuals. In a recent cross-cultural study the alpha coefficients for the independent and interdependent subscales of the Self-Construal Scale were different with low values for the Western group like those in the present study (62).

## Conclusion

In summary, the present study contributes to the literature by testing a mediation model which offers an understanding of the relationships between self-construal, PSMU and PSU, *via* the mediating role of the FoMO. The results indicated that interdependent self-construal could work as a risk factor mainly for PSMU rather than PSU. Results also suggested that FoMO may account for the previously established relationship not only between self-construal and PSMU but mainly for PSU. However, future studies should analyse and replicate the proposed model with other psychological variables. For instance, social comparison can be evaluated as a mediator in this relationship. It is well-established that for individuals with higher interdependent self-construal, other people become a source of the definition of the self, and social comparison may be used primarily when self-improvements motives are strong. Furthermore, previous studies have indicated that social comparison is positively associated with social media use and PSU because they seek comparison information (64, 65).

## Data Availability Statement

The raw data supporting the conclusions of this article will be made available by the authors, without undue reservation.

## Ethics Statement

The studies involving human participants were reviewed and approved by University of Calabria. The participants provided their written informed consent to participate in this study.

## Author Contributions

RS: conceived and designed the study, analyzed the data, and wrote the first draft of the paper. ZD, MG, and BK: reviewed and improved subsequent drafts of the paper. All authors have read and agreed to the published version of the manuscript.

## Conflict of Interest

The authors declare that the research was conducted in the absence of any commercial or financial relationships that could be construed as a potential conflict of interest.

## Publisher's Note

All claims expressed in this article are solely those of the authors and do not necessarily represent those of their affiliated organizations, or those of the publisher, the editors and the reviewers. Any product that may be evaluated in this article, or claim that may be made by its manufacturer, is not guaranteed or endorsed by the publisher.

## References

[1] Billieux J Maurage P Lopez-Fernandez O Kuss DJ Griffiths MD. Can disordered mobile phone use be considered a behavioral addiction? An update on current evidence and a comprehensive model for future research. Curr Addict Rep. (2015) 2:156–62. 10.1007/s40429-015-0054-y

[2] Montag C Wegmann E Sariyska R Demetrovics Z Brand M. How to overcome taxonomical problems in the study of internet use disorders and what to do with “smartphone addiction?”. J Behav Addict. (2021) 9:908–14. 10.1556/2006.8.2019.59 31668089 PMC8969715

[3] Elhai JD Yang H Fang J Bai X Hall BJ. Depression and anxiety symptoms are related to problematic smartphone use severity in Chinese young adults: fear of missing out as a mediator. Addict Behav. (2020) 101:105962. 10.1016/j.addbeh.2019.04.020 31030950

[4] Li Y Li G Liu L Wu H. Correlations between mobile phone addiction and anxiety, depression, impulsivity, and poor sleep quality among college students: a systematic review and meta-analysis. J Behav Addict. (2020) 9:551–71. 10.1556/2006.2020.00057 32903205 PMC8943681

[5] Yang Z Asbury K Griffiths MD. An exploration of problematic smartphone use among Chinese university students: associations with academic anxiety, academic procrastination, self-regulation and subjective well-being. Int J Ment Health Addict. (2019) 17:596–614. 10.1007/s11469-018-9961-1

[6] Huang S Lai X Xue Y Zhang C Wang Y A network analysis of problematic smartphone use symptoms in a student sample. J Behav Addict. (2021) 9:1032–43. 10.1556/2006.2020.00098 33372911 PMC8969737

[7] Servidio R. Self-control and problematic smartphone use among Italian university students: the mediating role of the fear of missing out and of smartphone use patterns. Curr Psychol. (2021) 40:4101–11. 10.1007/s12144-019-00373-z

[8] Starri M. Global Digital Report 2021 - Italy. We Are Social. (2021) Available online at: https://wearesocial.com/it/blog/2021/02/digital-2021-i-dati-italiani/

[9] Busch PA McCarthy S. Antecedents and consequences of problematic smartphone use: a systematic literature review of an emerging research area. Comput Hum Behav. (2021) 114:106414. 10.1016/j.chb.2020.106414

[10] Canale N Moretta T Pancani L Buodo G Vieno A Dalmaso M. Test of the pathway model of problematic smartphone use. J Behav Addict. (2021) 10:181–93. 10.1556/2006.2020.00103 33475526 PMC8969864

[11] Balta S Jonason P Denes A Emirtekin E Tosuntaş SB Kircaburun K. Dark personality traits and problematic smartphone use: the mediating role of fearful attachment. Personal Individ Differ. (2019) 149:214–9. 10.1016/j.paid.2019.06.005

[12] Servidio R Griffiths MD Demetrovics Z. Dark triad of personality and problematic smartphone use: a preliminary study on the mediating role of fear of missing out. Int J Environ Res Public Health. (2021) 18:8463. 10.3390/ijerph18168463 34444212 PMC8391539

[13] Servidio R. Fear of missing out and self-esteem as mediators of the relationship between maximization and problematic smartphone use. Curr Psychol. (2021). 10.1007/s12144-020-01341-8

[14] Wang P Zhao M Wang X Xie X Wang Y Lei L. Peer relationship and adolescent smartphone addiction: the mediating role of self-esteem and the moderating role of the need to belong. J Behav Addict. (2017) 6:708–17. 10.1556/2006.6.2017.079 29254360 PMC6034960

[15] Casale S Fioravanti G Spada MM. Modelling the contribution of metacognitions and expectancies to problematic smartphone use. J Behav Addict. (2021) 10:788–98. 10.1556/2006.2021.00066 34613932 PMC8997219

[16] Marengo D Sindermann C Häckel D Settanni M Elhai JD Montag C. The association between the big five personality traits and smartphone use disorder: a meta-analysis. J Behav Addict. (2020) 9:534–50. 10.1556/2006.2020.00069 33011714 PMC8943667

[17] Starcevic V King DL Delfabbro PH Schimmenti A Castro-Calvo J Giardina A Billieux J. Diagnostic inflation” will not resolve taxonomical problems in the study of addictive online behaviors: commentary on: how to overcome taxonomical problems in the study of Internet use disorders and what to do with “smartphone addiction?. J Behav Addict. (2021) 9:915–9. 10.1556/2006.2020.00083 33289693 PMC8969725

[18] Dalvi-Esfahani M Niknafs A Kuss DJ Nilashi M Afrough S. Social media addiction: applying the DEMATEL approach. Telemat Inform. (2019) 43:101250. 10.1016/j.tele.2019.101250

[19] Ryan T Chester A Reece J Xenos S. The uses and abuses of Facebook: a review of Facebook addiction. J Behav Addict. (2014) 3:133–48. 10.1556/JBA.3.2014.016 25317337 PMC4189307

[20] Kircaburun K Demetrovics Z Tosuntaş SB. Analyzing the links between problematic social media use, dark triad traits, and self-esteem. Int J Ment Health Addict. (2019) 17:1496–507. 10.1007/s11469-018-9900-1

[21] Rozgonjuk D Kattago M Täht K. Social media use in lectures mediates the relationship between procrastination and problematic smartphone use. Comput Hum Behav. (2018) 89:191–8. 10.1016/j.chb.2018.08.003

[22] Kuss DJ Kanjo E Crook-Rumsey M Kibowski F Wang GY Sumich A. Problematic mobile phone use and addiction across generations: the roles of psychopathological symptoms and smartphone use. J Technol Behav Sci. (2018) 3:141–9. 10.1007/s41347-017-0041-3 30238057 PMC6133055

[23] Balta S Emirtekin E Kircaburun K Griffiths MD. Neuroticism, trait fear of missing out, and phubbing: the mediating role of state fear of missing out and problematic Instagram use. Int J Ment Health Addict. (2020) 18:628–39. 10.1007/s11469-018-9959-8

[24] Tandon A Dhir A Talwar S Kaur P Mäntymäki M. Social media induced fear of missing out (FoMO) and phubbing: behavioral, relational and psychological outcomes. Technol Forecast Soc Change. (2022) 174:121149. 10.1016/j.techfore.2021.121149

[25] Markus HR Kitayama S. Culture and the self: implications for cognition, emotion, and motivation. Psychol Rev. (1991) 98:224–53. 10.1037/0033-295X.98.2.224

[26] Singelis TM. The measurement of independent and interdependent self-construals. Pers Soc Psychol Bull. (1994) 20:580–91. 10.1177/0146167294205014

[27] Triandis HC Gelfand MJ. A theory of individualism and collectivism. Handbook of theories of social psychology. Sage Publications Ltd. (2012). 498–520 10.4135/9781446249222.n51

[28] Giacomin M Jordan C. Interdependent and independent self-construal. In: Zeigler-Hill V Shackelford TK editors. Encyclopedia of Personality and Individual Differences. Cham: Springer International Publishing (2017). p. 1–7. 10.1007/978-3-319-28099-8_1136-1

[29] Triandis HC. Individualism & Collectivism. Boulder: Westview Press. (1995).

[30] Dogan V. Why do people experience the fear of missing out (FoMO)? Exposing the link Between the self and the FoMO through self-construal. J Cross-Cult Psychol. (2019) 50:524–38. 10.1177/0022022119839145

[31] Kim JH Kim MS Nam Y. An analysis of self-construals, motivations, Facebook use, and user satisfaction. Int J Hum-Comput Interact. (2010) 26:1077–99. 10.1080/10447318.2010.516726

[32] Chen B Marcus J. Students' self-presentation on Facebook: an examination of personality and self-construal factors. Comput Hum Behav. (2012) 28:2091–9. 10.1016/j.chb.2012.06.013

[33] Ferenczi N Marshall TC Bejanyan K. Are sex differences in antisocial and prosocial Facebook use explained by narcissism and relational self-construal? Comput Hum Behav. (2017) 77:25–31. 10.1016/j.chb.2017.08.033

[34] Hawi N Samaha M. Identifying commonalities and differences in personality characteristics of Internet and social media addiction profiles: traits, self-esteem, and self-construal. Behav Inf Technol. (2019) 38:110–9. 10.1080/0144929X.2018.1515984

[35] Lin L Wang X Li Q Xia B Chen P Wang W. The influence of interpersonal sensitivity on smartphone addiction: a moderated mediation model. Front Psychol. (2021) 12:670223. 10.3389/fpsyg.2021.670223 34366988 PMC8339309

[36] Przybylski AK Murayama K DeHaan CR Gladwell V. Motivational, emotional, and behavioral correlates of fear of missing out. Comput Hum Behav. (2013) 29:1841–8. 10.1016/j.chb.2013.02.014

[37] Boustead R Flack M. Moderated-mediation analysis of problematic social networking use: the role of anxious attachment orientation, fear of missing out and satisfaction with life. Addict Behav. (2021) 119:106938. 10.1016/j.addbeh.2021.106938 33845255

[38] Fioravanti G Casale S Benucci SB Prostamo A Falone A Ricca V. Fear of missing out and social networking sites use and abuse: a meta-analysis. Comput Hum Behav. (2021) 122:106839. 10.1016/j.chb.2021.106839

[39] Brown L Kuss DJ. Fear of missing out, mental well-being, and social connectedness: a 7-day social media abstinence trial. Int J Environ Res Public Health. (2020) 17:4566. 10.3390/ijerph17124566 32599962 PMC7345987

[40] Zsido AN Arato N Lang A Labadi B Stecina D Bandi SA. The role of maladaptive cognitive emotion regulation strategies and social anxiety in problematic smartphone and social media use. Personal Individ Differ. (2021) 173:110647. 10.1016/j.paid.2021.110647

[41] Wang P Wang X Nie J Zeng P Liu K Wang J. Envy and problematic smartphone use: the mediating role of FOMO and the moderating role of student-student relationship. Personal Individ Differ. (2019) 146:136–42. 10.1016/j.paid.2019.04.013

[42] Markus HR Kitayama S. Cultures and selves: a cycle of mutual constitution. Perspect Psychol Sci. (2010) 5:420–30. 10.1177/1745691610375557 26162188

[43] Fang J Wang X Wen Z Zhou J. Fear of missing out and problematic social media use as mediators between emotional support from social media and phubbing behavior. Addict Behav. (2020) 107:106430. 10.1016/j.addbeh.2020.106430 32289745

[44] Li L Niu Z Mei S Griffiths MD. A network analysis approach to the relationship between fear of missing out (FoMO), smartphone addiction, and social networking site use among a sample of Chinese university students. Comput Hum Behav. (2022) 128:107086. 10.1016/j.chb.2021.107086

[45] Tandon A Dhir A Almugren I AlNemer GN Mäntymäki M. Fear of missing out (FoMO) among social media users: a systematic literature review, synthesis and framework for future research. Internet Res. (2021) 31:782–821. 10.1108/INTR-11-2019-0455

[46] Savci M Tekin A Elhai JD. Prediction of problematic social media use (PSU) using machine learning approaches. Curr Psychol. (2020) 10.1007/s12144-020-00794-1

[47] Elhai JD Levine JC Alghraibeh AM Alafnan AA Aldraiweesh AA Hall BJ. Fear of missing out: testing relationships with negative affectivity, online social engagement, and problematic smartphone use. Comput Hum Behav. (2018) 89:289–98. 10.1016/j.chb.2018.08.020

[48] Blackwell D Leaman C Tramposch R Osborne C Liss M. Extraversion, neuroticism, attachment style, and fear of missing out as predictors of social media use and addiction. Personal Individ Differ. (2017) 116:69–72. 10.1016/j.paid.2017.04.039

[49] Brand M Wegmann E Stark R Müller A Wölfling K Robbins TW. The interaction of person-affect-cognition-execution (I-PACE) model for addictive behaviors: update, generalization to addictive behaviors beyond internet-use disorders, and specification of the process character of addictive behaviors. Neurosci Biobehav Rev. (2019) 104:1–10. 10.1016/j.neubiorev.2019.06.032 31247240

[50] Dempsey AE O'Brien KD Tiamiyu MF Elhai JD. Fear of missing out (FoMO) and rumination mediate relations between social anxiety and problematic Facebook use. Addict Behav Rep. (2019) 9:100150. 10.1016/j.abrep.2018.100150 31193746 PMC6542373

[51] Brand M Young KS Laier C Wölfling K Potenza MN. Integrating psychological and neurobiological considerations regarding the development and maintenance of specific internet-use disorders: an interaction of person-affect-cognition-execution (I-PACE) model. Neurosci Biobehav Rev. (2016) 71:252–66. 10.1016/j.neubiorev.2016.08.033 27590829

[52] Aparicio-Martínez P Ruiz-Rubio M Perea-Moreno A-J Martínez-Jiménez MP Pagliari C Redel-Macías MD. Gender differences in the addiction to social networks in the Southern Spanish university students. Telemat Inform. (2020) 46:101304. 10.1016/j.tele.2019.101304

[53] Arpaci I. Gender differences in the relationship between problematic internet use and nomophobia. Curr Psychol. (2020) 16:e0250509. 10.1007/s12144-020-01160-x34003860

[54] D'Amico A Scrima F. The Italian validation of Singelis's self-construal scale (SCS): a short 10-item version shows improved psychometric properties. Curr Psychol. (2016) 35:159–68. 10.1007/s12144-015-9378-y

[55] Vaske JJ Beaman J Sponarski CC. Rethinking internal consistency in Cronbach's alpha. Leis Sci. (2017) 39:163–73. 10.1080/01490400.2015.1127189

[56] Casale S Fioravanti G. Factor structure and psychometric properties of the Italian version of the fear of missing out scale in emerging adults and adolescents. Addict Behav. (2020) 102:106179. 10.1016/j.addbeh.2019.106179 31704432

[57] Kwon M Kim DJ Cho H Yang S. The smartphone addiction scale: development and validation of a short version for adolescents. PLoS One. (2013) 8:e83558. 10.1371/journal.pone.0083558 24391787 PMC3877074

[58] Monacis L de Palo V Griffiths MD Sinatra M. Social networking addiction, attachment style, and validation of the Italian version of the Bergen social media addiction scale. J Behav Addict. (2017) 6:178–86. 10.1556/2006.6.2017.023 28494648 PMC5520120

[59] Andreassen CS Pallesen S Griffiths MD. The relationship between addictive use of social media, narcissism, and self-esteem: findings from a large national survey. Addict Behav. (2017) 64:287–93. 10.1016/j.addbeh.2016.03.006 27072491

[60] De Pasquale C Sciacca F Hichy Z. Italian validation of smartphone addiction scale short version for adolescents and young adults (SAS-SV). Psychology. (2017) 08:1513–8. 10.4236/psych.2017.810100

[61] Muthén LK Muthén BO. Mplus (Version 7.2) [Computer Software]. CA:Los Angeles: Muthén & Muthén (2014).

[62] Huang CM Doole R Wu CW Huang HW Chao YP. Culture-related and individual differences in regional brain volumes: a cross-cultural voxel-based morphometry study. Front Hum Neurosci. (2019) 13:313. 10.3389/fnhum.2019.00313 31551740 PMC6746838

[63] Servidio R Sinatra M Griffiths MD Monacis L. Social comparison orientation and fear of missing out as mediators between self-concept clarity and problematic smartphone use. Addict Behav. (2021) 122:107014. 10.1016/j.addbeh.2021.107014 34153569

[64] Song H Cramer EM Park N. Cultural differences in social comparison on facebook. Behav Inf Technol. (2019) 38:172–83. 10.1080/0144929X.2018.1519037

[65] Chu S-C Windels K Kamal S. The influence of self-construal and materialism on social media intensity: A study of China and the United States. Int J Advert. (2016) 35:569–88. 10.1080/02650487.2015.1068425

